# Prognostic Factors in the Surgery of Clinoidal Meningiomas

**DOI:** 10.7759/cureus.40122

**Published:** 2023-06-08

**Authors:** Cristopher Valencia-Ramos, Jose G Arroyo Del Castillo, Jorge F Aragon, Aldo G Eguiluz-Mendez, Gerardo Y Guinto-Nishimura, Marcos V Sangrador-Deitos, Juan Luis Gómez Amador

**Affiliations:** 1 Neurosurgery, Instituto Nacional de Neurología y Neurocirugía Manuel Velasco Suárez, Mexico City, MEX

**Keywords:** outcome, pronostic factors, surgery, meningioma, clinoid

## Abstract

Introduction: Clinoidal meningiomas are currently among the intracranial meningiomas with the greatest neurosurgical complexity, morbidity, and mortality associated with their resection. The worldwide literature has described tumor size (>4 cm^3^), patient age (>60 years), and invasion of the cavernous sinus as factors associated with a worse clinical outcome following surgery.

Methods: We describe the series of cases of patients with clinoidal meningiomas who underwent microsurgical resection at our institution between January 2014 and March 2019. The intention was to analyze the multiple preoperative variables, such as the patient's demographic, tumor, and surgical characteristics, such as the Al-Mefty Classification to find a relationship with the patient's clinical outcome during their postoperative follow-up.

Results: Death occurred in 4.8% of the cases. Postoperative morbidity was documented in 42.9%, the most frequent being ophthalmoparesis, followed by worsening of visual acuity, and new onset motor deficits. Radiological characteristics were assessed based on preoperative MRI. The maximum diameter, midline shift, invasion of the cavernous sinus, arterial encasement, and peritumoral edema were evaluated. Average intraoperative bleeding was 1.3 L. The most frequent histological grade was World Health Organization (WHO) grade 1 in 85.6% of the cases. Complete resection was obtained in 52.4% of the cases; 42.8% received fractionated stereotactic radiotherapy after surgery for disease control, and one received radiosurgery. Recurrence occurred in 33.3%. The average follow-up in months was 23.8.

Conclusions: Demographic factors and tumor characteristics in clinoidal meningioma surgery are related to the subtype of meningioma according to the Al-Mefty Classification and impact directly the degree of resection, progression of the disease, and degree of postoperative complications. To achieve maximal resection while minimizing morbidity and mortality, these factors must be considered to decide on an appropriate approach and specific plan for each case.

## Introduction

Meningiomas originate from the arachnoid's cap cells, and surgery represents the mainstay for treatment [1]. According to the statistical analysis of the Central Nervous System (CNS) Tumor Registry of the United States of America, in 2018, meningiomas were the most common CNS tumors, accounting for 37.1% [2]. Clinoidal meningiomas are part of the so-called sphenoid wing meningiomas, from which they account for 34%-44.9% of these lesions [3-4]. 

Clinoidal meningiomas are described as those meningiomas growing in the vicinity of the anterior clinoid process [5]. They were first described in 1910 by Frotscher and Becker [6]. In 1938, Cushing and Eisenhardt described them as deep meningiomas of the sphenoid crest or the clinoidal third. It was not until 1990 that Al-Mefty coined the term “clinoidal meningiomas” and classified them into three types depending on their growth pattern and relationship to the anterior clinoid process. Type 1 is defined as those that grow from the inferior part of the clinoid process and are intimately related to the internal carotid artery; type 2 originates from the superior and lateral part of the clinoid process; and type 3 originates from the optic canal on the medial side of the anterior clinoid process [7]. In 2010, Pamir and Belirgen proposed a modification to this classification by adding the size of the tumor in its coronal diameter. Group A includes those less than 2 cm, Group B from 2 to 4 cm, and Group C (giants) includes those measuring more than 4 cm [3].

The neurosurgical complexity of this type of meningioma lies in the relationship that the anterior clinoid process has with multiple neurovascular structures. The oculomotor nerve runs along its anterolateral aspect, the carotid artery is in close proximity to its inferior aspect, and the optic nerve lies along its anteromedial aspect [1, 3-5]. The main clinical scenario in this type of tumor is headache and visual disturbances. Other symptoms are usually related to the size and extension of the tumor, for example, tumors that extend to the cavernous sinus may be associated with cranial neuropathies. Some cases may extend to the middle fossa compressing the temporal lobe, leading to seizures, or even compressing the cerebral peduncle, causing hemiparesis. Some cases may present with proptosis and orbital pain secondary to hyperostosis in the orbital region [3-5]. Visual symptoms may vary and most are due to optic nerve atrophy. Some syndromes have been rarely described, such as cavernous sinus, orbital apex, and Foster-Kennedy syndromes [3-7].

From a surgical perspective, there are two main issues that must be addressed when approaching these lesions. First, it is of paramount importance to design the best approach for each tumor. Second, deciding which is the best management strategy for the intracavernous portion of the tumor if present [6, 8-10]. Since clinoidal meningiomas tend to grow dorsally, true invasion of the cavernous sinus is rare, with rates of invasion ranging from 0 to 44.1% [3]. In a meta-analysis that includes 1208 patients, complete resection was achieved in 64.2% (95% CI, 57.3%-71%), with rates of 11.8% for Al-Mefty group I, 92.6% for group II, and 84.2% for group III [11]. Reported recurrence rates after incomplete resection range from 4% to 15% [6-7, 12-13]. Mortality associated with surgery in these tumors ranges from 0 to 15.4% [11, 14-18]. 

In the last decades, neurosurgical morbidity and mortality have significantly decreased. However, clinoidal meningiomas remain among the tumors with the highest surgical complications and recurrence rates within intracranial meningiomas [6]. Since they may present as large tumors, they are likely to adhere to more distal vessels such as the middle cerebral artery and anterior cerebral artery making them prone to vascular injuries, especially true Al-Mefty's group I meningiomas [19]. The postoperative visual decline may be due to direct damage or vascular injury to the optic nerve or chiasm. Other reported postoperative complications include meningitis, hydrocephalus, and new-onset seizures [3, 11, 14]. Overall, a postoperative visual improvement of 48% has been observed [11].

Within the case series reported in the literature on clinoidal meningiomas and the clinical outcome of patients undergoing microsurgical resection, the following preoperative predictive factors have been found: tumor size, patient age, invasion of the cavernous sinus, and previous visual deficit [3, 6-7, 10-17, 19-27]. The objective of this article is to describe the relationship between tumor and patient characteristics, as well as the percentage of resection and clinical outcome of patients with clinoidal meningiomas in a third-level institution in Latin America.

## Materials and methods

We conducted a retrospective review of all the patients diagnosed with clinoidal meningiomas surgically treated at our center between January 2014 and March 2019. Data collected from the clinical and radiological records of these patients were used, taking into account epidemiological and demographic data, risk factors, functional status (Karnofsky Performance Score [KPS]) before and after the procedure, modified Rankin Scale (mRS), preoperative neurological symptoms, type of neurosurgical approach, tumor characteristics (size by volumetry, consistency, vascular encasement, invasion of the cavernous sinus, invasion of the optic canal, and Al-Mefty classification), percentage of resection, Simpson classification, and procedure-associated morbidity and mortality. 

The main inclusion criteria were as follows: patients of any gender and age who have undergone surgery for clinoidal meningioma resection at the National Institute of Neurology and Neurosurgery between January 2014 and March 2019, patients with preoperative and postoperative enhanced MRI within a period of fewer than three months, those who have accepted and signed informed consent for anterior clinoidal meningioma resection surgery, and patients with a minimum follow-up of one month after surgery. Patients not meeting the inclusion criteria or with incomplete clinical records were excluded. 

The main clinical outcome results were measured using the KPS, which were related to operative bleeding, tumor volume, invasion of the cavernous sinus, maximum diameter in centimeters, peritumoral edema, pre-surgical visual or cranial nerve deficits, arterial encasement, the extent of resection, residual tumor, recurrence, presence of vascular complications, presence of temporary and permanent neurological complications.

Operational definitions

The degree of resection was classified according to postoperative imaging studies (MRI images). All pathological personal history was taken from the clinical record. The initial presenting symptom was defined as the one that prompted the patient to seek medical attention. Surgical bleeding was defined as estimated by the anesthesiology service and reported in the medical record. Complications were recorded based on what was reported in the clinical record. Demographic and clinical variables of the population were documented. Continuous variables were summarized as means or medians and categorical variables were reported as percentages.

Univariate analysis was performed to evaluate the quality of the collected data, including missing and complete data, descriptive statistics with measures of central tendency and dispersion, presented as percentages for nominal variables, median and range for ordinal variables, and mean and standard deviation for dimensional variables. Normality tests (Kolmogorov-Smirnov) were conducted to determine whether the distribution of numerical variables was normal or non-normal.

Correlations between variables were calculated using Pearson correlation and logistic regression for continuous and binary variables, respectively. A statistically significant p-value was considered to be <0.05. The statistical analysis was conducted using Statistics Package for Social Sciences (SPSS) v21 (IBM Corp., Armonk, NY).

## Results

Patients’ characteristics

Forty-two patients were included in the study. The population comprised 17 males (40.5%) and 25 females (59.5%). The mean age was 49.7 ± 12.5 years (range: 26-74 years). The included meningiomas were divided into three groups according to the Al-Mefty classification, with eight cases classified as type 1 (19%), 30 as type 2 (71.5%), and four as type 3 (9.5%). Three patients (7.1%) had undergone previous embolization. Three other patients (7.1%) had previous surgery at another institution, and one (2.4%) of these received adjuvant treatment with radiotherapy. Of the total, 19 (45.2%) were taking steroids prior to their surgical procedure. 

Clinical characteristics

The most common initial symptom was a mild progressive headache in 20 patients (47.6%), followed by neurological focalization in 12 (28.6%), seizures, generalized tonic-clonic seizures in eight patients (19%), and finally acute intracranial hypertension in two patients (4.8%). The average time from symptom onset to surgical procedure was 33.2 months with a range of 0.5-430, and a median of 5.5 months. Among the focal neurologic signs, 36 patients (85.7%) had visual acuity and/or campimetry alterations, and 12 (28.6%) had motor deficits. The functional status of the patients was evaluated using the Karnofsky index; only four patients (9.5%) underwent surgery with a low functional status (<70), while the rest (90.5%) had a good functional status (>70).

The death occurred in two patients (4.8%). Postoperative morbidity was documented in 18 patients (42.9%), the most frequent being post-surgical ophthalmoparesis in 12 (28.6%), followed by worsening of visual acuity after surgery in nine (21.4%), and five (12%) presented new-onset motor deficits. Among the most frequent post-surgical complications were post-surgical infarction in three patients (7.1%), and surgical site hematoma that did not require further intervention in three (7.1%). Only one patient (2.4%) required re-intervention due to postsurgical hematoma. Three patients (7.1%) had to be left without bone flap due to transoperative brain swelling, only one (2.4%) presented surgical site infection, and two (4.8%) presented internal cerebrospinal fluid fistula (Table 1).

**Table 1:** Demographic and clinical characteristics. SD, standard deviation; ICH, intracranial hypertension; KPSS, Karnofsky performance status scale

Demographic and clinical characteristics			
Variable		n	Percentage (%)
Gender	Male	17	40.5
	Female	25	59.5
	Total	42	100
The average age in years (range) 49.7 (26-74) SD 12.5			
Al-Mefty grade	Type 1	8	19
	Type 2	30	71.5
	Type 3	4	9.5
Medical history	Previous stent embolization	3	7.1
	Previous surgery	3	7.1
	Radiotherapy	1	2.4
	Preoperative steroid	19	45.2
Initial symptoms	Headache	20	47.6
	Neurological focalization	12	28.6
	Seizures	8	19
	ICH	2	4.8
KPSS categorized prior to surgery	< 70	4	9.5
	≥ 70	28	90.5

Radiological characteristics

Meningiomas’ radiological characteristics were assessed based on preoperative MRI studies. The maximum diameter was evaluated as the variable for meningioma size. The average maximum diameter was 5.28 cm, with a standard deviation of 1.37 and a range of 2.7-8.2 cm. Thirty-six (85.7%) presented midline shift, 24 (57.1%) had invasion of the cavernous sinus, 27 (64.3%) had arterial encasement, and 33 (78.6%) had peritumoral edema.

Histological grade, Simpson grade of resection, and functional clinical outcome

The most frequent surgical approach was the pterional approach, performed in 30 patients (71.4%), followed by the orbito-cranial approach in seven (16.6%), and the orbito-zygomatic approach in five (12%). The average intraoperative bleeding was 1.3 L (SD 0.7 L) with a range of 0.3-3 L. The most frequent histological grade was WHO grade 1 in 32 patients (85.6%), followed by grade 2 in five (12%), and grade 3 in only one (2.4%). Total gross resection was evaluated according to the Simpson classification, achieving grade 1 in 11 patients (26.2%), grade 2 in eight (19%), grade 3 in three (7.14%), and grade 4 in 20 (47.6%). In the strict sense of the Simpson classification, grades 1, 2, and 3 represent a complete resection of the meningioma, therefore a complete resection was obtained in our series in 22 patients (52.4%). Eighteen patients (42.8%) received fractionated stereotactic radiotherapy after surgery for disease control, and only one received radiosurgery (2.4%). Recurrence occurred in 14 patients (33.3%), of whom only three (7.1%) had not received adjuvant radiotherapy during follow-up, and five (12%) required new surgical intervention due to tumor recurrence during follow-up.

Functional status was evaluated using the Karnofsky score (Table 2). The worsening or improvement of the functional status was defined as a decrease or increase, respectively, of >20 points in the functional index. 

**Table 2:** Morbidity and clinical outcomes. SSI, surgical site infection; CSF fistula, cerebrospinal fluid fistula; KPSS, Karnofsky performance status

Morbidity and clinical outcomes			
Variable		n	Percentage (%)
Morbidity	Total	18	42.9
	Ophthalmoparesis	12	28.6
	Motor weakness	5	12
	Visual deficit	9	21.4
	Infarction/Ischemia	3	7.1
	Hematoma	4	9.5
	Second surgery	1	2.4
	SSI	1	2.4
	CSF fistula	2	4.8
Mortality		2	4.8
Clinically stable		29	69
Clinical improvement		5	12
Clinical worsening		8	19
KPSS categorized at 3 months after surgery	KPSS <70	12	28.5
	KPSS ≥ 70	30	71.5

Clinical stability was considered as a variation within 20 points of their baseline functional status. An unfavorable functional outcome was considered as a score <70. As mentioned earlier, only four patients were admitted to the operating room with poor functional status, of which only one improved and the rest remained the same. At the end of their follow-up, 12 patients (28.5%) had an unfavorable functional outcome (KPS <70). When evaluating changes in their preoperative and postoperative functional status, only eight patients (19%) worsened, and 5 (12%) showed improvement after surgery. The remaining 29 patients (69%) maintained a similar functional status to their presurgical state. The average follow-up period in months was 23.8 (SD 14.6; 3-51), and a median of 26.5. 

Mortality and morbidity by subgroup

The global mortality was 4.76% (two patients) and the overall morbidity was 42.85% (18 patients). In Table 3, we describe the specific morbimortality by subtype according to the Al-Mefty classification. The majority of morbidity occurred in patients with subtype 2, as it was the most frequent type in the series. 

Regarding type 1 cases, two patients had postoperative ophthalmoparesis, one had new motor deficits, two had new visual deficits, and one had postoperative brain infarction and subsequently died of nosocomial pneumonia. 

For patients with type 2 meningiomas, there was morbidity in 11 patients, new ophthalmoparesis in 8, a new pyramidal syndrome in 3, added visual deficit in 6, postoperative cerebral infarction secondary to severe vasospasm in 2, one had to be re-intervened due to neurological deterioration secondary to a hematoma in the surgical field, one had surgical site infection, and two had internal cerebrospinal fluid fistulae which were treated conservatively. For Al-Mefty type 3 clinoidal meningiomas, two patients had secondary morbidity, both had postoperative ophthalmoparesis, and one had pyramidal syndrome and postoperative visual deterioration. There was no mortality in this subgroup as seen in Table 3.

**Table 3:** Post-procedure morbidity by Al-Mefty classification. SSI, surgical site infection; CSF fistula, cerebrospinal fluid fistula

Post-procedure morbidity by Al-Mefty classification			
Al-Mefty classification	Type 1	Type 2	Type 3
Mortality/subtype total (%)	1/8(12.5)	1/30(3.3)	0/4
Morbidity/subtype total (%)	5/8(62.5)	11/30(36.6)	2/4(50)
Ophthalmoplegia (%)	2(25)	8(26.7)	2(50)
Motor weakness (%)	1(12.5)	3(10)	1(25)
Visual deficit (%)	2(25)	6(20)	1(25)
Infarct/ischemia (%)	1(12.5)	2(6.7)	0
Re-intervention (%)	0	1(3.3)	0
SSI (%)	0	1(3.3)	0
CSF fistula (%)	0	2(6.7)	0

Procedures performed in the hybrid operating room

Combined procedures were performed in the hybrid operating room by endovascular therapy and neurosurgery, simultaneously. The three patients (7.14%) underwent microsurgical resection of a clinoidal meningioma after placement of a stent in the distal portion of the internal carotid artery and proximal portion of the middle cerebral artery, with intraoperative angiography guided by "roadmapping" technique. Two of these patients had an unfavorable clinical outcome according to the Karnofsky index (<60), due to postoperative stent occlusion with secondary cerebral infarction, while the remaining patient showed significant clinical improvement. Complete tumor resection was achieved in all three patients, with a Simpson grade 1 in one patient and grade 2 in the other two patients.

Illustrative case

**Figure 1:**
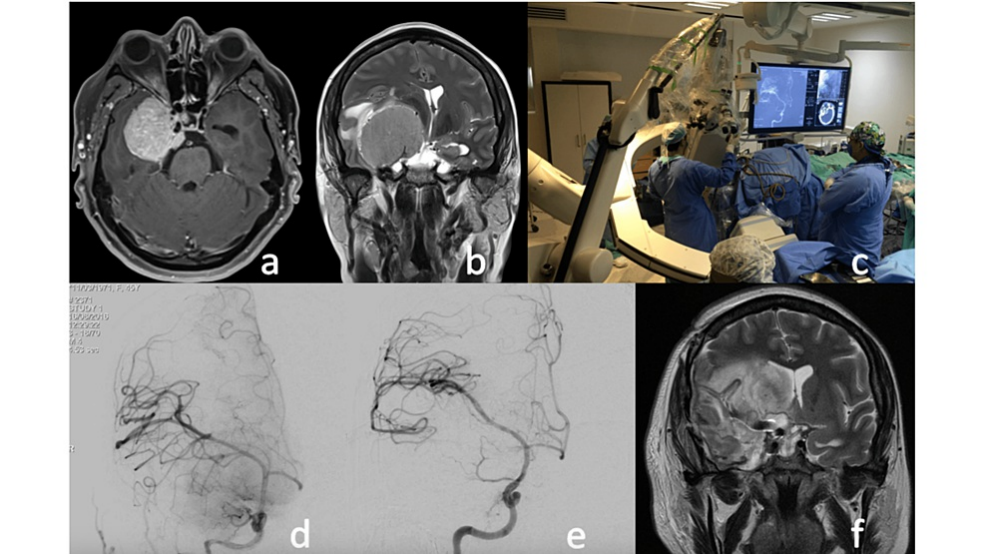
Illustrative case. The patient underwent resection of a type 1 Al-Mefty clinoidal meningioma with the prior placement of a stent in the middle cerebral artery and internal carotid artery guided by roadmapping. a. Axial SPGR-Gd brain MRI showing a hyperintense homogeneous lesion surrounding the ipsilateral internal carotid artery to the meningioma, b. Coronal T2 MRI showing an isointense lesion surrounding the ipsilateral middle cerebral artery and causing mass effect. c. Hybrid operating room, roadmap during the resection with transoperative angiography, d and e. Digital subtraction angiography of the right carotid artery before and after the procedure. f. Coronal T2 MRI without evidence of the lesion, with the presence of hyperintensity in the surgical bed, cerebral edema causing displacement of the midline structures contralaterally.

A 44-year-old female with a history of 6 months of visual acuity deficits in the right eye and chronic progressive headache underwent a brain MRI, which showed a right cerebral neoplasm involving the distal segments of the internal carotid artery, the sphenoidal segment of the middle cerebral artery, and the ipsilateral optic nerve (Figure 1). Surgical resection in a hybrid operating room was planned, with the prior placement of a stent in the mentioned vascular segments. Complete transcranial resection was performed through a right pterional approach, with dissection of the ipsilateral cavernous sinus/middle fossa, and no vascular injury was observed. The histopathological report confirmed a transitional meningioma. Unfortunately, the patient had postoperative embolic events in the middle cerebral artery territory that resulted in an unfavorable functional state.

## Discussion

Over the years, neurosurgical procedures have evolved with the advent of tools that assist the neurosurgeon during complex procedures. This has been reflected in the better surgical outcomes for most of the neurosurgical diseases. Clinoidal meningiomas are no exception to this rule, with morbidity and mortality rates decreasing from the first descriptions to the present day. Nonetheless, these tumors continue to be related to a high incidence of morbidity. 

Since its inception, our institution has been a reference in the field of neuroscience in our country and Latin America. Hence, the evolution in the surgical treatment of brain tumors and the outcome of neurological patients has improved over the years. Therefore, the series of clinoidal meningiomas over a five-year period is a reflection of a disease described in the global literature, with results comparable to those previously described [9, 11-13]. 

The objective of this study is to report the experience of a third-level neuroscience center in the treatment of clinoidal meningiomas and their prognostic factors and clinical outcomes, in order to describe their results and compare them with those of international literature [9, 11-13]. Due to the sample size, no relationship was found between treatment and diagnosis with the clinical outcome of patients, that is, in their functional status according to their Karnofsky score three months after surgery, either jointly or according to their subtype, with a non-significant p-value. Most studies in the literature related to the treatment of clinoidal meningiomas are series or case reports, however, there are some exceptions [11-13]. Giammattei et al. reported a case series with a meta-analysis of prognostic factors in clinoidal meningioma surgery, where predictive factors for an unfavorable outcome in this type of pathology are age over 70 years, a maximum lesion diameter greater than 4 cm, invasion of the cavernous sinus, peritumoral edema, and visual or oculomotor nerve deficit prior to the procedure [11].

Due to the selection bias of these patients, there is no equivalence of the general data with international reports for the presented tumors, such as gender distribution in any of the cases, which was higher for females, tumor size, and symptom presentation, which are biased due to being a tertiary institution and do not represent the representative data of a sample of the general population. Patients who are candidates for appropriate treatment and approach for this type of tumor, by expert consensus, will continue to be added to this case series in order to have a more precise statistical analysis of their therapeutic behaviors and functional outcome.

Preoperative embolization can help in the resection of skull base meningiomas by reducing the blood supply to the tumor, making it easier to remove during surgery. This is because meningiomas are vascular tumors that receive their blood supply from arteries that originate from various parts of the skull base. By blocking these arteries with embolic agents, the blood flow to the tumor can be reduced, which can decrease intraoperative bleeding and facilitate surgical removal. However, it is important to note that preoperative embolization is not without risk and should be carefully considered on a case-by-case basis. We consider that some clinoidal meningiomas might benefit from this therapeutic approach [28-30].

Hybrid OR brain tumors

Within this report, three patients presented with primary brain neoplasms, specifically clinoidal meningiomas. In 2017, Al-Mefty et al. published a case series of giant brain tumors in which both embolization and resection were performed in a hybrid operating room with excellent results [28]. In the case of the three patients with lesions involving the internal carotid artery and the M1 portion of the ipsilateral middle cerebral artery, both neurosurgery and endovascular therapy services reached a consensus to perform stent placement over the distal portion of the internal carotid artery and proximal portion of the middle cerebral artery where the lesion was located, in order to provide protection and better vascular control during resection in the hybrid surgery procedure.

Unfortunately, the functional status of both patients was low during their 3-month follow-up, but this may be due to two causes. The first patient had an embolic event a couple of days after stent placement, which conditioned an ischemic vascular event that led to a low preoperative mRS. The second patient developed a postsurgical hematoma which required a second exploratory surgery, with hematoma drainage and decompressive hemicraniectomy, which significantly decreased her functional status. Despite the above, complete resection of the meningioma was performed on all three patients during the hybrid procedure, without any vascular injury noted on transoperative angiographies or immediately after the procedure. That is to say, if it were not for the external events to the hybrid procedure that are described as complications of both neurosurgery and endovascular therapy procedures in the worldwide literature, the functional status of the patients probably would not have been affected [27-28].

Hybrid operating room in our center

These are the first reported cases with the formal implementation of a hybrid operating room protocol with the joint and simultaneous management of neurosurgical microsurgery and endovascular therapy treatments in Latin America. There has been some previous experience in the hybrid operating room for the treatment of brain aneurysms [30]. 

This series has been the result of hard work to address the needs of a complex pathology that undoubtedly challenges any treatment modality and demonstrates some of the strategies that have evolved over time for the management of these lesions. It could even be considered that the integration of well-established endovascular and microsurgical therapy is a safe practice for patients. However, randomized multicenter clinical trials with adequate methodology are necessary to demonstrate if this is actually true.

Worldwide, there are few healthcare centers with adequate facilities to perform this type of procedure, and even in developing countries like ours, hospitals that perform these procedures are scarce. Currently, it is difficult to define whether these joint procedures in a hybrid operating room are more effective than each separately. However, it can be considered a reality that these combined treatments offer a wider response in a single event by a larger group of specialists focused on the treatment of complex neurovascular diseases.

Limitations

The study conducted is descriptive. With a total of 42 patients, although within the average of reported series worldwide, the number of patients is small. There was no control group to compare the results obtained in the series, according to the type of clinoidal meningioma according to the Al-Mefty classification and the microsurgical approach, so the statistical analysis does not have enough power to draw definitive conclusions regarding the risk factors related to this type of pathology by subtype. Despite the limitations mentioned, the study achieved its objective, as it describes in detail the results obtained in one of the largest series of clinoidal meningiomas in Latin America, as well as their prognostic factors for clinical outcome. One of the strengths of the study is that more than half of the patients have a follow-up of more than 24 months after the procedure, allowing for the assessment of their long-term evolution, as well as the recurrence rate. A larger cohort, including a clinical trial in which complex cases can be randomized for treatment, will allow us to validate the results obtained preliminarily in this case series.

Ethical considerations

The study ensures the bioethical aspects inherent in clinical research studies, such as confidentiality of the obtained information, the principle of autonomy of the participating subjects, the principle of beneficence and informed consent, without having conflicts of interest, and allowing access to source documents if requested by regulatory authorities.

The study is considered as research with minimal risk according to the regulation of the General Health Law regarding Health Research, given that it involves common procedures in physical or psychological diagnostic or routine treatment examinations. It also adheres to the guidelines established in the Helsinki Declaration.

## Conclusions

Demographic factors and tumor characteristics in clinoidal meningioma surgery at our center are related to the subtype of meningioma according to the Al-Mefty Classification as described in other international series and impact directly the degree of resection, progression of the disease, and degree of postoperative complications. To achieve maximal resection while minimizing morbidity and mortality, these factors must be considered to decide on an appropriate approach and specific plan for each case. Worldwide clinoidal meningiomas continue to be a challenge for neurosurgeons due to their proximity to important neurovascular structures, such as the internal carotid artery and its branches, the optic nerve, and the oculomotor nerve. 

## References

[1] Ayoubi S, Dunn IF, Al-Mefty O (2011). Meningiomas. Brain Tumors.

[2] Ostrom QT, Gittleman H, Truitt G (2018). CBTRUS statistical report: primary brain and other central nervous system tumors diagnosed in the United States in 2011-2015. Neuro Oncol.

[3] Pamir MN, Belirgen M (2010). Chapter 29 - Anterior Clinoidal Meningiomas. Elsevier Inc.

[4] Guinto G (2012). Surgical Management of Sphenoid Wing Meningiomas. Vol 6. 6th ed..

[5] Krisht AF (2011). Al-Mefty’s meningiomas. Al-Mefty's Meningiomas. 2nd ed.

[6] Bassiouni H, Asgari S, Sandalcioglu IE (2009). Anterior clinoidal meningiomas: functional outcome after microsurgical resection in a consecutive series of 106 patients. Clinical article. J Neurosurg.

[7] Al-Mefty O (1990). Clinoidal meningiomas. J Neurosurg.

[8] Lee JH, Sade B, Park BJ (2006). A surgical technique for the removal of clinoidal meningiomas. Neurosurgery.

[9] Kanaan INI (2004). Sphenoid wing meningiomas. Int Congr Ser.

[10] Yoshimoto K, Nakamizo A, Sasaki T (2013). Surgical techniques for the dissection of encased perforators in giant clinoidal meningiomas. Acta Neurochir (Wien).

[11] Giammattei L, Starnoni D, Levivier M (2019). Surgery for clinoidal meningiomas: case series and meta-analysis of outcomes and complications. World Neurosurg.

[12] Pamir MN, Belirgen M, Ozduman K (2008). Anterior clinoidal meningiomas: analysis of 43 consecutive surgically treated cases. Acta Neurochir (Wien).

[13] Puzzilli F, Ruggeri A, Mastronardi L (1999). Anterior clinoidal meningiomas: report of a series of 33 patients operated on through the pterional approach. Neuro Oncol.

[14] Salgado López L, Muñoz Hernández F, Asencio Cortés C (2018). Extradural anterior clinoidectomy in the management of parasellar meningiomas: analysis of 13 years of experience and literature review. Neurocirugia (Astur : Engl Ed).

[15] Chernov SV, Rzaev DA, Kalinovsky AV (2017). [Early postoperative results of surgical treatment of patients with anterior clinoidal meningiomas]. Zh Vopr Neirokhir Im N N Burdenko.

[16] Attia M, Umansky F, Paldor I (2012). Giant anterior clinoidal meningiomas: surgical technique and outcomes. J Neurosurg.

[17] Romani R, Laakso A, Kangasniemi M (2011). Lateral supraorbital approach applied to anterior clinoidal meningiomas: experience with 73 consecutive patients. Neurosurgery.

[18] Güdük M, Özduman K, Pamir MN (2019). Sphenoid wing meningiomas: surgical outcomes in a series of 141 cases and proposal of a scoring system predicting extent of resection. World Neurosurg.

[19] Salunke P, Singh A, Kamble R (2019). Vascular involvement in anterior clinoidal meningiomas: biting the 'artery' that feeds. Clin Neurol Neurosurg.

[20] Cui H, Wang Y, Yin YH (2007). Surgical management of anterior clinoidal meningiomas: a 26-case report. Surg Neurol.

[21] Nagata T, Ishibashi K, Metwally H (2013). Analysis of venous drainage from sylvian veins in clinoidal meningiomas. World Neurosurg.

[22] Sade B, Lee JH (2008). High incidence of optic canal involvement in clinoidal meningiomas: rationale for aggressive skull base approach. Acta Neurochir (Wien).

[23] Kim JH, Jang WY, Jung TY (2017). Predictive factors for surgical outcome in anterior clinoidal meningiomas: analysis of 59 consecutive surgically treated cases. Medicine (Baltimore).

[24] Czernicki T, Kunert P, Nowak A (2015). Results of surgical treatment of anterior clinoidal meningiomas - our experiences. Neurol Neurochir Pol.

[25] Mariniello G, de Divitiis O, Seneca V (2012). Classical pterional compared to the extended skull base approach for the removal of clinoidal meningiomas. J Clin Neurosci.

[26] Nanda A, Konar SK, Maiti TK (2016). Stratification of predictive factors to assess resectability and surgical outcome in clinoidal meningioma. Clin Neurol Neurosurg.

[27] Lee JH, Jeun SS, Evans J (2001). Surgical management of clinoidal meningiomas. Neurosurgery.

[28] Almefty RO, Patel NJ, See AP (2017). Hybrid surgery management of giant hypervascular tumors: intraoperative endovascular embolization with microsurgical resection. World Neurosurg.

[29] Yoon N, Shah A, Couldwell WT (2023). Preoperative embolization of skull base meningiomas: current indications, techniques, and pearls for complication avoidance. Neurosurg Focus.

[30] Gómez-Amador JL, Valencia-Ramos CG, Sangrador-Deitos MV (2023). Roadmapping technique in the hybrid operating room for the microsurgical treatment of complex intracranial aneurysms. J Cerebrovasc Endovasc Neurosurg.

